# Characteristics, Epigenetics, and Management of Non-Infectious Preterm Birth—Sterile Intrauterine Inflammation and Idiopathic Preterm Birth

**DOI:** 10.3390/life16060882

**Published:** 2026-05-25

**Authors:** Vilmos Fulop, László Kalmár, György Végh, Sándor Nagy, Borbála Szeiler, Kornél Lakatos

**Affiliations:** 1 Faculty of Health Sciences, University of Miskolc, 3515 Miskolc, Hungary; 2 Department of Obstetrics and Gynecology, Northern Pest Central Hospital Military Hospital, 1134 Budapest, Hungary; 3 Department of Obstetrics and Gynecology, Northern Buda Szent János Hospital, 1125 Budapest, Hungary; 4 Department of Obstetrics and Gynecology, Szechenyi University, 9026 Gyor, Hungary; 5 Department of Perinatal Intensive Care Unit, Northern Pest Central Hospital Military Hospital, 1134 Budapest, Hungary

**Keywords:** sterile intrauterine inflammation, preterm birth, epigenetics

## Abstract

Preterm birth is a major cause of neonatal morbidity and mortality, and many spontaneous cases remain idiopathic. Increasing evidence suggests that intrauterine inflammation may occur in the absence of detectable infection, leading to the recognition of sterile intrauterine inflammation as an important mechanism contributing to threatened preterm labor and spontaneous preterm birth. This review summarizes current knowledge regarding the role of damage-associated molecular patterns (DAMPs), alarmins, pattern recognition receptors, inflammasome activation, cellular senescence, and pyroptosis in the initiation of sterile inflammatory pathways associated with labor. Key mediators including HMGB1, IL-1α, fetal cell-free DNA, platelet-activating factor, and S100 proteins appear to promote inflammatory activation within fetal membranes and the amniotic cavity. The review also discusses the emerging contribution of fetal immune activation, maternal–fetal immune dysregulation, maternal microchimerism, and epigenetic mechanisms to idiopathic preterm birth. Current diagnostic and therapeutic options remain limited, and no targeted treatment for sterile intrauterine inflammation has yet been established. Future approaches may include precision biomarkers, multiomics-based risk stratification, targeted immunomodulatory therapies, and modulation of maternal–fetal immune interactions. Improved understanding of sterile inflammatory mechanisms may ultimately support development of personalized strategies to prevent preterm birth and improve perinatal outcomes.

## 1. Introduction

During pregnancy, the maternal immune system must maintain a delicate equilibrium between protective immune responses and tolerance toward the fetus. Threatened preterm labor and preterm birth are multifactorial conditions that may arise spontaneously or result from medical intervention. Numerous risk factors for preterm birth have been extensively investigated, including multiple gestation, previous preterm delivery, low socioeconomic status, medication or substance use, short intervals between pregnancies, infections, and chronic maternal conditions such as diabetes and hypertension. Nevertheless, in many cases, no definitive cause can be identified, and these cases are classified as idiopathic preterm births.

Infection and inflammation are responsible for approximately 30–50% of preterm births [1]. Inflammatory processes, mediated by both pro-inflammatory and anti-inflammatory cytokines, contribute to the progression of labor, beginning with uterine contractions and advancing through cervical ripening to membrane rupture and delivery.

Traditionally, intrauterine inflammation has been considered a consequence of maternal immune responses to microorganisms invading the amniotic cavity [2,3,4]. However, several clinical studies have demonstrated that some pregnant women with evidence of intrauterine inflammation—most commonly identified by elevated interleukin-6 (IL-6) levels in amniotic fluid—show negative amniotic fluid cultures. This observation has led to two possible explanations: either the inflammation is caused by microorganisms that cannot be cultured using conventional methods, or it arises from noninfectious mechanisms similar to those involved in sterile inflammatory conditions such as gout or rheumatoid arthritis [5].

Over the past two decades, advances in molecular microbiology have improved the detection of previously non-cultivable microorganisms in amniotic fluid. Consequently, the combined use of standard culture techniques with molecular diagnostic approaches, including PCR-ESI/MS and assays for microbial cell-free DNA (cfDNA), has enabled confirmation that some cases of intrauterine inflammation occur in the absence of detectable bacterial or viral nucleic acids. This entity has therefore been termed “sterile intrauterine inflammation” [6]. Among women with intact membranes who present with asymptomatic ultrasound-detected cervical shortening or cervical insufficiency, sterile intrauterine inflammation appears to be more common than intrauterine infection [7]. Importantly, pregnancy complications and neonatal outcomes associated with sterile intrauterine inflammation resemble those observed in infection-related intrauterine inflammation. Clinically, sterile intrauterine inflammation is also associated with acute placental inflammatory lesions, including acute histologic chorioamnionitis (HCA) and funisitis, findings similarly observed in microbial intrauterine inflammation. Sterile intraamniotic inflammation, defined as inflammation occurring without identifiable microorganisms or infection, is a relatively recently recognized condition. In the past decade, inflammatory biomarkers have proven valuable in the diagnosis and management of various gynecological disorders [8]. Although generally less intense than infection-induced inflammation, sterile inflammation likewise promotes activation of neutrophils, monocytes, macrophages, and CD4+ T lymphocytes, together with increased expression of inflammatory cytokines [9].

## 2. Methodology

This review was conducted using a structured literature search strategy to identify studies investigating sterile intrauterine inflammation, sterile intraamniotic inflammation, fetal immune activation, inflammasome signaling, maternal–fetal immune interactions, and epigenetic mechanisms associated with threatened preterm labor and spontaneous preterm birth.

A comprehensive search of the PubMed, Scopus, and Google Scholar databases was performed for articles published in English and Hungarian up to 2026. Search terms included combinations of the following keywords: sterile intrauterine inflammation, sterile intraamniotic inflammation, preterm birth, preterm labor, DAMPs, alarmins, HMGB1, inflammasome, NLRP3, fetal immune activation, maternal–fetal tolerance, microchimerism, epigenetics, and chorioamnionitis. Original research articles, clinical studies, experimental studies, and relevant review articles were included. Studies primarily focused on infection-associated preterm birth without discussion of sterile inflammatory mechanisms were excluded unless they provided important comparative data. Priority was given to recent publications, landmark studies, and articles addressing molecular and immunological mechanisms relevant to sterile inflammation and idiopathic spontaneous preterm birth. The retrieved literature was reviewed narratively, and findings were synthesized according to major thematic areas including innate immune activation, alarmins and DAMPs, inflammasome signaling, fetal immune responses, epigenetic regulation, and emerging therapeutic approaches.

## 3. Main Characteristics and Pathophysiology of Non-Infectious Preterm Birth and Sterile Intrauterine Inflammation

Sterile intraamniotic inflammation (SIAI) and sterile intrauterine inflammation (SIUI) represent noninfectious inflammatory conditions driven primarily by damage-associated molecular patterns (DAMPs), alarmins, and inflammasome activation [10]. SIAI is localized to the amniotic cavity, whereas SIUI is a broader process involving multiple intrauterine compartments, including the decidua, placenta, fetal membranes, and amniotic cavity. Both conditions are closely associated with idiopathic spontaneous preterm birth (isPTB), suggesting that sterile inflammation may trigger labor even in the absence of detectable infection. Fetal immune activation further links these disorders, as activation of fetal dendritic cells, T cells, and inflammatory cytokine pathways may amplify intrauterine inflammation and contribute directly to the initiation of preterm labor. Chorioamnionitis without detectable infection represents the histopathological manifestation of this sterile inflammatory process within the fetal membranes and is frequently associated with SIUI, SIAI, fetal immune activation, adverse perinatal outcomes, and neonatal and long-term effects (See Table 1). Even children born from asymptomatic pregnancies with sterile inflammation may be at increased risk of later neurodevelopmental disorders [11]. Similar to infection, sterile inflammation can cause pulmonary (BPD), brain (IVH), or intestinal complications in the newborn. Human conception, followed by the division and implantation of the fertilized egg into the maternal decidua, is a complex process that requires the coordinated functioning of embryological, immunological processes and anatomical structures. The steps of these processes are genetically determined and the coarse- and fine-tuning of these effects occurs at the epigenetic level by external effects such as intrauterine environment, hormones, toxins, changes in diet, infections, or cell differentiation. Disturbances of these mechanisms for example by sterile intrauterine inflammation are the basis of several pathologies that threaten conception, implantation and successful pregnancy, such as recurrent miscarriage, preeclampsia, fetal growth restriction or premature birth. In addition, epigenetic alterations acting during the period of plasticity of individual development can cause diseases that appear in the young, adult and even old age of the offspring, which can even be hereditary through generations. The gene expression of immune and tumor cells is regulated by epigenetic processes. These processes can enhance the immune system evasion mechanisms of various tumor cells (see Table 1).

### 3.1. DAMPs, PAMPs, and Pattern Recognition Receptors in Labor

Damage-associated molecular patterns (DAMPs) and pathogen-associated molecular patterns (PAMPs), through their interaction with pattern recognition receptors (PRRs), play a central role in triggering the inflammatory cascade that contributes to both normal term labor and preterm labor. During pregnancy, Toll-like receptors (TLRs), which represent a major subgroup of PRRs, may become activated even in the absence of infection, thereby promoting sterile inflammation through the action of DAMPs [12].

**Figure 1:**
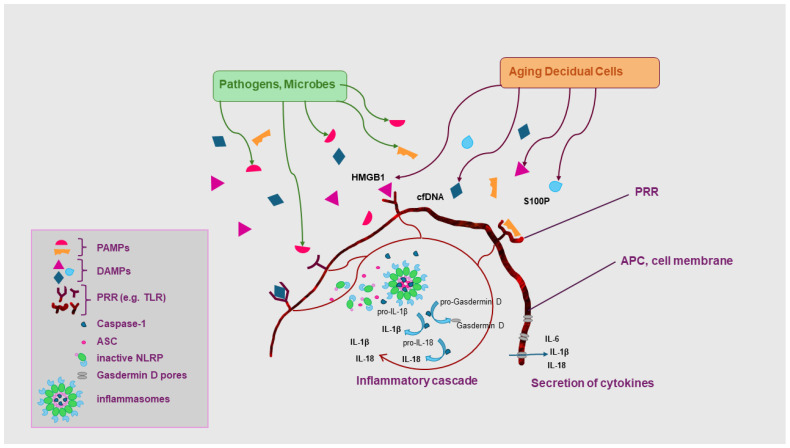
Intrauterine inflammation induced by alarmins. Alarmins (PAMPs, DAMPs) play a fundamental role in initiating the inflammatory cascade. These exogenous and endogenous danger signals are detected by certain cellular elements of the innate immune system (APCs: macrophages, dendritic cells) via membrane-bound and cytosolic pattern-recognition receptors. Sterile intrauterine inflammation is an inflammatory response triggered not by infection but by endogenous danger signals. These DAMPs activate the inflammatory cascade through PRRs—including TLRs—and this process contributes both to labor at term and to threatened preterm birth. In amniotic fluid during sterile inflammation, IL-1α and HMGB1 accumulate particularly, and their concentrations correlate with the risk of preterm birth. Alarmins can activate inflammasomes, which leads to the maturation of proinflammatory cytokines and to pyroptotic cell death, further amplifying inflammation. ASC specks detectable in amniotic fluid are markers of inflammasome activation and are also elevated in sterile preterm birth. Aging of the fetal membranes, oxidative stress, and cell damage cause further DAMP release, which activates the common final pathway of labor. HMGB1, S100 proteins, IL-1α, HSP70, cell-free DNA, and PAF are all alarmins whose levels have been shown to increase in threatened preterm birth. Abbreviations: PAMP—pathogen-associated molecular pattern; DAMP—damage-associated molecular pattern; APC—antigen-presenting cell; PRR—pattern recognition receptor; TLR—Toll-like receptor; IL—interleukin; HMGB1—high mobility group box 1; ASC—inflammasome adaptor protein; HSP70—heat shock protein 70; cfDNA—cell-free DNA; PAF—platelet-activating factor; NLRP—NOD-like receptor protein.

Among the DAMPs most frequently investigated in threatened preterm labor and uterine contractions associated with term delivery are high-mobility group box-1 protein (HMGB1), fetal cell-free DNA (cfDNA), and platelet-activating factor (PAF) (See Figure 1).

### 3.2. Alarmins and Sterile Intrauterine Inflammation

To better differentiate inflammatory responses associated with sterile intrauterine inflammation from those related to intrauterine infection, early network analyses evaluated cytokines and inflammatory mediators present in the amniotic fluid of pregnant women with threatened preterm labor and intact membranes. These studies demonstrated increased accumulation of IL-1α and HMGB1 in the amniotic fluid of women with sterile intrauterine inflammation.

Numerous studies have subsequently confirmed the important role of IL-1α in both physiological and pathological labor processes [13]. Elevated concentrations of IL-1α have been identified in the amniotic fluid of women with intrauterine inflammation, and experimental studies in mice have shown that systemic administration of IL-1α can induce preterm birth.

Similarly, women with sterile intrauterine inflammation and higher amniotic fluid HMGB1 concentrations delivered earlier than those with lower levels, suggesting that HMGB1 contributes to threatened preterm labor and preterm birth. Both IL-1α and HMGB1 are recognized as classic DAMPs, or alarmins, emphasizing the central role of alarmins in the pathophysiology of sterile intrauterine inflammation [14].

Alarmins belong to a larger group of signaling molecules known as danger signals, which activate innate and adaptive immune responses and initiate host defense mechanisms. Exogenous danger signals originating from microorganisms are referred to as pathogen-associated molecular patterns (PAMPs), whereas endogenous signals released from host tissues are termed DAMPs or alarmins [14]. Emerging evidence further suggests that alarmins can be released during cellular senescence, a state of irreversible cell-cycle arrest. Well-characterized alarmins include HMGB proteins, members of the S100 protein family, IL-1α, and heat-shock protein 70 (HSP70) [15].

### 3.3. Cellular Senescence and Release of DAMPs

Oxidative stress and senescence of the fetal amnion and chorion stimulate the release of DAMPs, which subsequently initiate labor-associated inflammatory pathways and uterine contractions. Senescence of fetal membrane tissues is considered part of the normal physiological process of labor, whereas decidual senescence has been implicated as a distinct mechanism contributing to noninfectious spontaneous preterm birth.

Consequently, senescent cells may represent an important source of alarmins in the amniotic fluid of women with threatened preterm labor and sterile intrauterine inflammation.

In senescent cells, DAMPs translocate from the nucleus into the cytoplasm, from where they can be secreted as alarmins and trigger inflammatory pathways leading to labor [16]. Experimental studies have demonstrated that intraamniotic administration of HMGB1 induces preterm birth in mice, while elevated HMGB1 concentrations have been consistently observed in the amniotic fluid of women with threatened preterm labor, regardless of the presence of infection, further implicating HMGB1 in sterile intrauterine inflammation [17,18].

Importantly, increased concentrations of several alarmins have been reported in the amniotic fluid of women presenting with threatened preterm labor and in those delivering preterm in the setting of intrauterine inflammation [19]. These findings support a direct association between elevated amniotic fluid alarmin levels, preterm birth, and adverse neonatal outcomes.

### 3.4. Inflammasome Activation in Term and Preterm Labor

Inflammasomes are large cytoplasmic protein complexes that regulate inflammatory responses [20]. Once activated, inflammasomes induce autocatalytic cleavage of pro-caspase-1 into its active form, which subsequently processes pro-IL-1β and pro-IL-18 into mature inflammatory cytokines that play crucial roles in both term and preterm labor [21]. Inflammasome activation also leads to formation of intracellular ASC “specks” by the adaptor protein ASC. These extracellular ASC specks can themselves function as alarmins, and their detection reflects in vivo inflammasome activation. Increased ASC concentrations have been documented in the amniotic fluid of women delivering at term [22], as well as in preterm patients with sterile intrauterine inflammation, supporting the hypothesis that alarmins can activate inflammasome pathways within the amniotic cavity. Exposure to HMGB1 has been shown to upregulate inflammatory mediators in fetal membrane tissues, including NLR family components such as NLRP3 and NOD1/2 at both mRNA and protein levels. PRRs such as NLRP3 and NOD2 activate inflammasomes, which are believed to contribute to the initiation of spontaneous labor at term and during preterm birth. Activation of these pathways during physiological labor induces IL-1β secretion in fetal membranes [23]. Placental PRR expression therefore acts as a critical defense mechanism capable of recognizing both microbial invasion and cellular stress. The terminal step of inflammasome activation is pyroptosis, a highly inflammatory form of programmed cell death characterized by gasdermin D (GSDMD)-mediated pore formation in the cell membrane, allowing release of cytosolic contents, including IL-1 family cytokines, into the extracellular space [24].

### 3.5. HMGB1 and Other DAMPs in Fetal Membrane Activation

Activation of fetal membrane tissues is recognized as part of the common pathway leading to labor. HMGB1 is predominantly expressed by amniotic epithelial cells, myofibroblasts, neutrophils, and macrophages [18]. Incubation of fetal membranes with HMGB1 stimulates release of proinflammatory cytokines such as IL-1β and IL-6 and enhances expression of inflammatory mediators including NF-κB1, TNF, IL-1α, IFN-γ, as well as HMGB1 receptors RAGE and TLR2, thereby amplifying inflammatory responses within the fetal membranes [25]. Fetal cell-free DNA (cfDNA), another important DAMP and activator of Toll-like receptors, is released during cellular death and activates placental TLR signaling pathways. Platelet-activating factor (PAF), an inflammatory phospholipid, also represents a critical DAMP involved in labor. Elevated PAF concentrations have been identified in the amniotic fluid of women with threatened preterm labor, and intrauterine administration of PAF induces preterm birth in mice through macrophage TLR signaling pathways [26].

**Figure 2:**
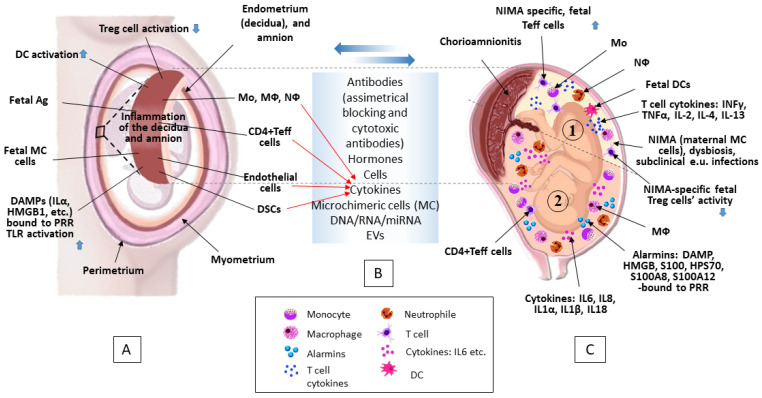
Preterm birth in sterile and idiopathic intrauterine inflammation. (**A**) During pregnancy and labor, TLRs expressed on immune and endothelial cells at the fetal–maternal interface can be activated during sterile inflammation by DAMPs, even in the absence of active infection. As delivery approaches, the decidual immune environment is characterized by interactions among various cell types, including macrophages, monocytes, and T cells, as well as decidual endothelial and stromal cells. (**B**) Examples include bidirectional transport of key signaling molecules and cells, such as hormones, cytokines, nucleic acids, extracellular vesicles (EVs), and microchimeric fetal and maternal cells. These molecules and cells influence both the maternal and fetal immune environments. Fetal microchimeric (MC) cells in the decidua can serve as a source of fetal antigens. These cells respond to increasing inflammatory signals mediated by DAMPs and cytokines, thereby triggering an inflammatory immune response that can initiate labor. In preterm birth, various components of fetal–maternal cross-talk may become dysregulated, leading to premature activation of inflammatory pathways. (**C**) This can occur when fetal–maternal tolerance breaks down, resulting in activation of maternal T cells against fetal antigens or fetal T cells against maternal antigens. (**①**) The fetal immune system itself can contribute to sterile threatened preterm birth and a subset of idiopathic preterm births, as indicated by the activation and increased presence of fetal T cells and their mediators in amniotic fluid. Reduced fetal Treg activity may permit activation of fetal antigen-specific Teff cells. Contributing factors may include nonhereditary maternal antigens (NIMA; e.g., maternal microchimeric cells), maternal dysbiosis, and subclinical infections at maternal extrauterine or other body sites. This immune response may also involve activation of innate immune cells present in the amniotic fluid, such as neutrophils and monocytes/macrophages. (**②**) Sterile intrauterine inflammation can be initiated by endogenous alarmins and is associated with increased concentrations of inflammatory mediators, including IL-6, IL-1α, IL-1β, and IL-18, together with infiltration of immune cells such as neutrophils, monocytes/macrophages, and T cells.

DAMPs released by stressed cells interact with PRRs, and increased levels of IL-1α and S100 family proteins, including calgranulin A (S100A8) and calgranulin C (S100A12), have been observed in the amniotic fluid during sterile intraamniotic inflammation [27,28,29] (Figure 2).

### 3.6. Clinical Significance of Sterile Intrauterine Inflammation and Maternal- Fetal Immune Tolerance During Pregnancy

Taken together, these findings establish sterile intrauterine inflammation as a distinct clinical condition associated with adverse perinatal outcomes. The inflammatory process is primarily triggered by alarmins, highlighting their central role in disease pathogenesis. Placental injury and dysfunction may consequently modify placental responses to both PAMPs and DAMPs, thereby affecting the course and outcome of pregnancy. DAMPs are not only important mediators of physiological term labor but also contribute directly to the development of threatened preterm labor in the context of sterile inflammation.

In healthy pregnancies, several interconnected mechanisms work together to preserve immune tolerance at the maternal–fetal interface. Maternal alloreactive T cells are prevented from crossing the placenta, while their activation is suppressed through expansion of regulatory T cells (Treg). In addition, uterine dendritic cells (DCs) remain localized and do not migrate to draining lymph nodes where they could otherwise activate maternal effector T cells (Teff).

Decidual B cells further contribute to immune tolerance by limiting inflammatory responses, including through production of asymmetric blocking antibodies. Another important mechanism involves the relative immaturity of fetal antigen-presenting cells (APCs) [30].

Despite these tolerance mechanisms, fetal T cells capable of recognizing nonhereditary maternal antigens (NIMA) are present even in uncomplicated pregnancies. Exposure to maternal microchimeric cells crossing the placenta can induce differentiation of these fetal T cells into Treg cells. Since measurable numbers of maternal microchimeric cells enter the fetal circulation during pregnancy, they may influence the timing and initiation of labor.

### 3.7. Fetal Immune Activation in Threatened Preterm Labor

Inflammation represents a systemic or localized tissue response mediated by a broad range of immune cells and soluble inflammatory mediators in response to infection or tissue injury. Although fetal T cells were previously considered immunologically immature and hyporesponsive, later studies demonstrated that fetal T cells within lymphoid organs, cord blood, and the fetal intestine are capable of mounting active immune responses [31].

Supporting this concept, both maternal and fetal immune cells participate in intrauterine inflammatory responses. In threatened preterm labor, inflammatory activation within the fetal compartment may initiate a cascade involving early activation of fetal dendritic cells and sensitization of fetal T cells to maternal antigens. Dysregulated fetal immune activation, together with increased production of inflammatory cytokines such as IFN-γ and TNF-α, may subsequently promote uterine contractions and trigger labor [30].

Increased activation of fetal DCs and T cells in cord blood from preterm infants has also been associated with higher numbers of maternal microchimeric cells. Furthermore, correlations between maternal microchimerism and central memory T cells suggest that maternal microchimeric cells may provide maternal antigens capable of activating alloreactive fetal Teff cells, thereby contributing to threatened preterm labor [30,32].

These findings indicate that activation of the fetal adaptive immune system, previously considered immature, may play a role in disruption of maternal–fetal tolerance in pregnancies complicated by threatened preterm labor.

### 3.8. Evidence for Fetal Immune Participation in Preterm Labor

Comparative studies of term and preterm labor have demonstrated activation of fetal immune cells in cord blood obtained before delivery by cordocentesis. Importantly, only a subset of preterm infants showed evidence of microbial exposure, suggesting that the fetus itself may generate immune responses sufficient to initiate labor.

A distinct population of central memory Th1 cells was identified in cord blood from preterm, but not term, neonates [30]. Previous investigations also identified several immune cell populations in microbe-free amniotic fluid that vary throughout gestation, including fetal innate lymphoid cells (ILCs) with intraepithelial-like phenotypes, suggesting tissue residency and responsiveness to intrauterine inflammation [33].

T lymphocytes constitute the predominant leukocyte population in preterm pregnancies and appear to be primarily of fetal origin based on DNA fingerprinting analyses. Increased numbers of fetal CD4+ T cells have been identified in the amniotic fluid of idiopathic preterm births. Similar to findings in ILCs, fetal T cells express markers associated with mucosal tissues, supporting the hypothesis that they originate from tissue compartments rather than the circulation [34,35].

In idiopathic preterm birth, fetal CD4+ T cells isolated from amniotic fluid express activation-associated cytokines such as IL-2, IL-4, and IL-13, indicating an active immune response. Moreover, compared with term neonates, T cells from the cord blood of infants born following idiopathic threatened preterm labor and preterm delivery exhibit enhanced immune responsiveness, further supporting a relationship between fetal T-cell activation and idiopathic preterm labor and birth. Activated fetal T cells may therefore directly contribute to induction of preterm birth, representing a novel pathogenic mechanism in idiopathic preterm labor [36]. The transfer of maternal microchimeric cells carrying nonhereditary maternal antigens may also influence reproductive fitness in subsequent generations. Through epigenetic and immunological mechanisms, maternal microchimerism acquired during pregnancy may affect long-term health and reproductive outcomes in female offspring, including susceptibility to pregnancy complications such as threatened preterm labor [37].

In addition to nonhereditary maternal antigens, other antigenic stimuli may contribute to fetal T-cell activation. Some women with threatened preterm labor exhibit dysbiosis or subclinical extrauterine infections capable of triggering inflammatory responses.

Bacterial products and pathogen-associated molecular patterns (PAMPs) stimulate production of proinflammatory cytokines that enhance fetal dendritic cell activation and promote differentiation of alloreactive T cells. Similarly, sterile inflammation mediated by DAMPs may also contribute to abnormal fetal T-cell activation [38].

## 4. Epigenetic Effects in Sterile Intrauterine Inflammation

Reduced progesterone signaling is a key factor driving the transition of the myometrium from a quiescent state to an active contractile state during labor. In addition to the myometrium, the endometrium and decidua also play critical roles in determining the timing of parturition. Decidual quiescence is actively established early in pregnancy and is characterized by transcriptional silencing within decidual stromal cells (DSCs), accumulation of the repressive histone marker H3K27me3, and epigenetic regulation of numerous genes involved in suppression of Th1-mediated immunity and wound-healing pathways [39]. Continuous progesterone signaling from conception onward is essential for maintaining this decidual quiescence and the anti-inflammatory immune environment required to sustain pregnancy until term labor.

Prenatal maternal stress has long been associated with elevated circulating levels of proinflammatory cytokines. Although preconception stress was not significantly linked to increased expression of proinflammatory genes or altered activity of transcription factors such as NF-κB or AP-1 [40], women experiencing high stress during pregnancy demonstrated greater expression of proinflammatory genes compared with those reporting lower stress levels.

Maternal exposure to chronic stress during childhood has also been associated with elevated maternal IL-6 levels, a recognized predictor of preterm birth. The same study further demonstrated that individuals with adverse childhood experiences were more likely to deliver preterm infants [41].

Another investigation examined disturbances in immune regulation and glucocorticoid resistance among socioeconomically disadvantaged pregnant women. In low-risk populations, cortisol concentrations negatively correlated with the ratio of proinflammatory to anti-inflammatory cytokines. This association was absent among socioeconomically disadvantaged high-risk women, suggesting impaired maternal stress responses and glucocorticoid resistance [42,43].

Sterile intraamniotic inflammation represents a relatively recent area of investigation with multiple proposed etiologies. One extensively studied mechanism involves dysregulation of the hypothalamic–pituitary–adrenal (HPA) axis as a potential contributor to sterile intrauterine inflammation.

Epigenetic alterations affecting inflammatory pathways and HPA axis–related genes have been evaluated in infants exposed or unexposed to intrauterine chorioamnionitis [44]. These data originated from an epigenome-wide association study analyzing peripheral blood samples from 157 infants. Researchers generated two gene panels: one consisting of 82 genes involved in the glucocorticoid receptor regulatory network associated with HPA axis function, and another related to regulation of cytokine production during inflammatory responses.

This exploratory analysis identified nine significantly differentially methylated CpG regions, including FKBP5, EP300, GATA3, GSK3B, IL6R, NFATC1, NR3C1, SMARCA4, and HIF1AN (adjusted *p*-values < 0.1).

Among 55 cytokine-related genes examined in infants born to mothers with or without chorioamnionitis, two CpG sites demonstrated significant differential methylation. One was located within the interleukin-6 receptor gene (IL6R). The biological significance of methylation depends on genomic location: promoter methylation is typically associated with gene silencing, whereas gene-body methylation generally correlates with active gene transcription. In this study, IL6R methylation occurred within the gene body, suggesting enhanced IL6R expression in infants exposed to chorioamnionitis compared with those without intrauterine inflammation [45].

The second cytokine-related gene identified was HIF1AN (Hypoxia Inducible Factor-1 Subunit Alpha Inhibitor), which is involved in several cellular processes, including negative regulation of the NOTCH signaling pathway that governs cellular proliferation and differentiation [46]. Promoter hypermethylation commonly results in transcriptional silencing [45]. Infants exposed to intrauterine chorioamnionitis demonstrated increased methylation within the HIF1AN promoter region. Potential suppression of HIF1AN expression may alter NOTCH pathway activity and thereby affect cell proliferation and differentiation during the critical developmental period encompassing the first 1000 days of life.

Further analysis of genes involved in glucocorticoid receptor regulation and HPA axis function identified statistically significant differential methylation in seven genes. Two CpG sites within FKBP5 showed higher methylation levels in infants without sterile chorioamnionitis. FKBP5 is a major regulator of HPA axis activity and glucocorticoid receptor sensitivity [47]. Increased promoter methylation may indicate reduced FKBP5 expression, potentially affecting glucocorticoid receptor feedback regulation. Another significantly altered CpG site was identified in the promoter region of NR3C1, the gene encoding the glucocorticoid receptor. Increased methylation of NR3C1 was observed in infants exposed to sterile chorioamnionitis, suggesting possible alterations in stress-response signaling pathways.

Collectively, these findings suggest potential differences in HPA axis regulation between infants exposed and unexposed to sterile chorioamnionitis, particularly at the level of DNA methylation. Comparisons between preterm and term infants also demonstrated increased methylation beta values in CpG sites associated with FKBP5, EP300, GSK3B, and NR3C1. Since the functional consequences of methylation depend on the specific genomic location, these observations may reflect dysregulation of HPA axis activity, particularly given the central role of FKBP5 in modulating glucocorticoid receptor sensitivity to cortisol.

## 5. Treatment of Sterile Intrauterine Inflammation

Sterile inflammatory conditions are generally managed with anti-inflammatory agents such as nonsteroidal anti-inflammatory drugs (NSAIDs) and corticosteroids. In some disorders, including gout, therapies aimed at reducing concentrations of alarmins that initiate sterile inflammation have also been utilized. However, many medications used to treat sterile inflammatory diseases are not approved for use during pregnancy. Consequently, there is currently no established or approved treatment specifically targeting sterile intrauterine inflammation.

Given the important role of the NLRP3 inflammasome in the pathogenesis of sterile intrauterine inflammation, inhibition of NLRP3 activation has been proposed as a potential therapeutic strategy to improve perinatal outcomes. In experimental animal models, the selective NLRP3 inhibitor MCC950 significantly reduced rates of preterm birth and neonatal mortality [22]. Nevertheless, MCC950 has not been approved for use during pregnancy, and further studies are required to determine its safety profile.

Because IL-1β is a downstream product of inflammasome activation, pretreatment with indomethacin was shown to decrease uterine contractions in macaques exposed to intrauterine IL-1β administration. In clinical practice, however, indomethacin is recommended only before 32 weeks of gestation due to the risk of premature fetal ductus arteriosus closure, which limits its utility in preventing preterm birth. Similarly, pretreatment with dexamethasone or IL-10 reduced both uterine contractility and IL-1β-induced intrauterine inflammation in macaque models [48].

At present, no reliable predictive tools exist to identify pregnant women at increased risk for sterile intrauterine inflammation, making prophylactic interventions difficult to implement in clinical practice. For this reason, repurposing medications already approved for use during pregnancy currently represents the most practical therapeutic approach.

Considering the urgent need for effective treatment strategies, two widely used pregnancy medications—betamethasone and clarithromycin—have been investigated in the context of sterile intrauterine inflammation [49]. Experimental studies suggest that betamethasone may prevent preterm birth, although it does not appear to significantly reduce neonatal mortality.

Clarithromycin, a macrolide antibiotic commonly used in the treatment of infection-associated intrauterine inflammation in women with threatened preterm labor and intact membranes, also exhibits strong anti-inflammatory properties through modulation of NF-κB and AP-1 signaling pathways [50]. Among macrolide antibiotics, clarithromycin has particularly effective placental transfer. Recent studies have demonstrated that clarithromycin reduces amniotic fluid IL-6 concentrations in women with early preterm prelabor rupture of membranes (PPROM) before 37 weeks’ gestation, as well as in cases of sterile intrauterine inflammation [51]. It is important to state that both betamethasone and clarithromycin should be interpreted as suggesting potential therapeutic use beyond current evidence.

Experimental data further indicate that clarithromycin can prevent HMGB1-induced preterm birth by interfering with mechanisms involved in labor initiation, including dysregulated expression of contraction-associated proteins and inflammatory mediators within intrauterine tissues. In addition, clarithromycin improved neonatal survival by attenuating inflammatory responses in the placenta as well as in fetal organs including the lungs, gastrointestinal tract, liver, and spleen. However, despite these beneficial effects, clarithromycin did not completely reverse HMGB1-associated neonatal mortality.

Importantly, women at risk of preterm birth related to intrauterine inflammation or infection are often treated simultaneously with corticosteroids and antibiotics in clinical practice [52].

## 6. Future Directions in the Management of Sterile Intrauterine Inflammation, Idiopathic Spontaneous Preterm Birth, and Fetal Immune Activation

### 6.1. Precision Diagnostics and Biomarker Development

As far as the prediction and risk assessment of preterm birth are concerned, sterile inflammation occurs more frequently in preterm birth with intact membranes than does infectious inflammation. Mothers diagnosed with sterile inflammation deliver at a similar early gestational week as infected patients, and the rate of fetal complications is similarly high [53]. In asymptomatic women with a short cervix, the presence of sterile inflammation significantly increases the risk of spontaneous preterm birth before 34 weeks. In clinical practice, it is important to distinguish the two because treatment may differ: The current gold standard for diagnosing SIUI is amniocentesis (analysis of amniotic fluid), assessing high levels of proinflammatory cytokines (e.g., IL-6, IL-8) in the absence of positive cultures [53]. Elevated levels of interleukin-6 (IL-6) (defined as an amniotic fluid (AF) interleukin (IL)-6 concentration ≥ 2.6 ng/mL) or MMP-8 in amniotic fluid are often used to identify sterile inflammation. High levels of certain “alarm markers” (such as the HMGB1 protein—high AF concentration of HMGB1 (≥8.55 ng/mL) delivered earlier than those with low AF concentration of HMGB) in the presence of sterile inflammation may predict even earlier delivery [53].

A major future direction is the development of reliable biomarkers capable of distinguishing sterile intrauterine inflammation from infection-driven inflammation, physiological inflammatory signaling from pathological activation and women at highest risk of spontaneous preterm birth. Some of the potential biomarkers have already been mentioned above like alarmins (or “alarm makers”)/DAMPs (HMGB1, S100 proteins, cfDNA), inflammasome-associated markers (ASC specks, IL-1β, IL-18) but according to recent studies, extracellular vesicles and transcriptomic or proteomic profiles derived from maternal blood, cervicovaginal fluid, placental tissue, or amniotic fluid.

Future approaches will likely combine wider biological data analysis (multiomics) and longitudinal immune profiling to create individualized risk prediction models.

### 6.2. Targeted Immunomodulatory Therapies and Modulation of the Maternal- Fetal Interface

While antibiotics are standard for infection, their role in sterile inflammation is debated, as they are largely ineffective at stopping preterm labor if there is no underlying infection, sometimes even shortening the latency period. However, according to our experience antibiotic shock therapy (around the 16th and 24th weeks of pregnancy) with macrolide antibiotics like clarithromycin or azitromycin to prevent SIUI or to treat women with threatened preterm labor with intact membranes, may be successful because they exhibit strong anti-inflammatory properties [51]. Furthermore, clarithromycin has particularly effective placental transfer. Nevertheless, unnecessary antimicrobial use in SIUI should be avoided because it may generate gut dysbiosis, trigger monocytic cytokine pathways and therefore inadvertently shorten the pregnancy and accelerate preterm.

Emerging protocols leverage amniotic fluid analysis to categorize the severity of inflammation. For example, 17-alpha hydroxyprogesterone caproate effectively prolongs pregnancy by up to 4 weeks in mild SIUI. As to non-antibiotic treatments, clinical trials are opened for specialized therapies, such as IL-1 pathway modulators and omega 3 fatty acid supplements to curb localized tissue degradation.

Developing drugs that block “alarm signals” (DAMPs) or inflammatory pathways. Investigation into treatments that reduce IL-1β activity and utilize agents like exendin-4 (Ex4) to modulate macrophage polarization (inducing M2 polarization) shows potential in mitigating adverse neonatal outcomes [54]. Active research is focused on using maternal blood samples to measure acute-phase proteins and adhesion molecules, reducing the need for invasive amniocentesis and investigation into how sterile inflammation affects multiomic screening (combining proteomics, metabolomics, etc.) to identify specific molecular trajectories of sterile inflammation and uterine muscle contractility. Since senescent fetal membrane cells contribute to sterile inflammation, therapies targeting premature aging of membranes are under investigation. Research into blockers of S100A12/receptor for advanced glycation end products (RAGE) interactions is also in process [55,56].

### 6.3. Microbiome and Extracellular Vesicle Research

Deeper analysis of the microbiome and extracellular vesical research has been in the focus of obstetrics and gynecology in the past decade (e.g., relation between endometriosis and microbiome) [57]. Although these conditions are considered “sterile,” maternal dysbiosis and subclinical microbial signals may still contribute to inflammation. Future studies will likely investigate maternal gut and vaginal microbiome modulation, probiotic or microbiome-targeted therapies and interactions between microbial metabolites and immune activation. Extracellular vesicles (EVs) are emerging as important mediators of fetal–maternal communication and may serve both as biomarkers and therapeutic targets [58].

## 7. Conclusions

Sterile intrauterine inflammation has emerged as a distinct and clinically important mechanism contributing to threatened preterm labor and idiopathic spontaneous preterm birth. Accumulating evidence demonstrates that inflammatory activation within the intrauterine environment may occur in the absence of detectable infection and can be driven by endogenous danger signals, including damage-associated molecular patterns (DAMPs) and alarmins such as HMGB1, IL-1α, fetal cell-free DNA, and S100 proteins. Through activation of pattern recognition receptors, inflammasomes, and downstream cytokine pathways, these mediators initiate inflammatory cascades that closely resemble those observed in infection-associated labor.

Cellular senescence, oxidative stress, inflammasome activation, and pyroptosis appear to play central roles in amplifying sterile inflammatory responses within fetal membranes, decidual tissues, and the amniotic cavity. At the same time, increasing evidence suggests that disruption of maternal–fetal immune tolerance and activation of the fetal adaptive immune system contribute significantly to the pathophysiology of idiopathic preterm labor. Fetal dendritic-cell activation, alloreactive fetal T-cell responses to nonhereditary maternal antigens, maternal microchimerism, and altered cytokine production collectively indicate that the fetus is not merely a passive participant but may actively contribute to the initiation of labor.

The interaction between sterile inflammation, fetal immune activation, epigenetic regulation, and environmental influences such as maternal stress further highlights the complex and multifactorial nature of preterm birth. Epigenetic alterations involving inflammatory and hypothalamic–pituitary–adrenal axis–related genes may contribute not only to the initiation of labor but also to long-term developmental and immunological consequences in exposed offspring.

Despite substantial progress in understanding the mechanisms underlying sterile intrauterine inflammation, major diagnostic and therapeutic challenges remain. Currently available clinical tools are insufficient to reliably distinguish sterile inflammation from infection-driven intrauterine inflammation or to identify women at highest risk for idiopathic spontaneous preterm birth. Moreover, no targeted therapies specifically approved for sterile intrauterine inflammation currently exist.

Future research should therefore focus on the development of precision diagnostic biomarkers, including alarmins, inflammasome-associated mediators, extracellular vesicles, and transcriptomic or proteomic signatures. Advances in multiomics technologies and longitudinal immune profiling may enable individualized risk stratification and earlier identification of pathological inflammatory activation. In parallel, targeted immunomodulatory approaches directed against IL-1 signaling, HMGB1, NLRP3 inflammasome activation, oxidative stress, and dysregulated maternal–fetal immune interactions may provide novel therapeutic opportunities. Further investigation of the microbiome, extracellular vesicles, and senescence-associated pathways may also improve understanding of the mechanisms linking sterile inflammation with preterm birth.

In conclusion, sterile intrauterine inflammation represents a complex immunological disorder involving coordinated interactions between innate immunity, adaptive fetal immune activation, placental dysfunction, cellular senescence, and epigenetic regulation. Improved understanding of these mechanisms may ultimately enable the development of personalized diagnostic and therapeutic strategies aimed at reducing the global burden of preterm birth and improving both maternal and neonatal outcomes.

## Figures and Tables

**Table 1:** Main Characteristics of Sterile Intraamniotic Inflammation (SIAI), Sterile Intrauterine Inflammation (SIUI), Idiopathic spontaneous preterm birth (sPTB), Fetal immune activation (FIA), Chorioamnionitis without detectable infection.

Condition	Definition	Main Pathophysiology	Presence of Microorganisms	Key Biomarkers/Findings	Clinical Features	Pregnancy/Neonatal Outcomes
**Sterile intra-amniotic inflammation (SIAI)**	Inflammation within the amniotic cavity without detectable microorganisms	Activation of innate immune pathways by DAMPs/alarms (e.g., HMGB1, cfDNA, PAF), inflammasome activation	No detectable bacteria, viruses, or fungi by culture or molecular testing	Elevated amniotic fluid IL-6, IL-1β, HMGB1, ASC specks, S100 proteins	Threatened preterm labor, cervical shortening, uterine contractions	Preterm birth, fetal inflammatory response, neonatal morbidity similar to infection-associated inflammation
**Sterile intrauterine inflammation (SIUI)**	Noninfectious inflammatory response involving intrauterine tissues (amniotic, chorionic, decidua, placenta)	DAMP-mediated activation of PRRs/TLRs, oxidative stress, cellular senescence, inflammasome activation	Absent detectable microbial invasion	Increased IL-6, IL-1α, HMGB1, NLRP3 activation, inflammatory cytokines	May occur with intact membranes, cervical insufficiency, or idiopathic preterm labor	Adverse perinatal outcomes, placental inflammatory lesions, increased risk of spontaneous PTB
**Idiopathic spontaneous preterm birth (SPTB)**	Spontaneous preterm birth without identifiable cause such as infection, placental pathology, or maternal disease	Multifactorial; associated with sterile inflammation, fetal immune activation, stress-related and epigenetic mechanisms	Usually absent	Elevated inflammatory mediators, activated fetal T cells, altered epigenetic markers (FKBP5, NR3C1, IL6R)	Preterm contractions, cervical shortening, preterm labor without obvious etiology	Prematurity-related neonatal complications and long-term developmental sequelae
**Fetal immune activation (FIA)**	Activation of the fetal immune system in response to intrauterine inflammatory stimuli	Activation of fetal dendritic cells, T cells, cytokine production (IFN-γ, TNF-α), maternal microchimerism	May occur with or without infection	Activated fetal CD4+ T cells, central memory Th1 cells, elevated fetal cytokines	Often associated with threatened preterm labor and intrauterine inflammation	Fetal inflammatory response syndrome (FIRS), preterm delivery, neonatal inflammatory complications
**Chorioamnionitis without detectable infection**	Histologic or clinical chorioamnionitis in the absence of identifiable microorganisms	Sterile inflammatory activation of placental and fetal membrane tissues by alarmins/DAMPs	Negative microbiological testing	Acute histologic chorioamnionitis, funisitis, elevated IL-6, HMGB1	Maternal fever may be absent; can present with preterm labor or PPROM	Similar outcomes to infectious chorioamnionitis, including PTB and neonatal morbidity

## Data Availability

No new data were created or analyzed in this study.

## Abbreviations

**Abbreviation|Full name|Function: IL:** Interleukin (Family of cytokines; cell–cell communication, regulation of inflammation and immune responses); **NF-κB:** Nuclear Factor kappa-light-chain-enhancer of activated B cells (Transcription factor; regulates genes for inflammation, survival, and immune responses); **NOTCH signaling:** Notch signaling (Direct cell–cell signaling pathway; regulates cell fate, proliferation, and differentiation); **FKBP5 gene:** FKBP5 (FK506 binding protein 5) (Modulates glucocorticoid receptor (GR) sensitivity; regulates stress response); **EP300 gene:** EP300 (p300) (Histone acetyltransferase (HAT); modifies chromatin to regulate transcription); **GATA3 gene:** GATA3 (Zinc-finger transcription factor; key for cell differentiation and development (e.g., T cells)); **GSK3B gene:** GSK3B (Glycogen synthase kinase 3 beta) (Multifunctional kinase; involved in glucose metabolism, development, and apoptosis); **IL6R:** Interleukin-6 receptor (Receptor for IL-6; mediates inflammatory and immune signaling); **NFATC1 gene:** NFATC1 (Nuclear factor of activated T-cells, cytoplasmic 1) (Transcription factor; regulates T-cell activation and differentiation); **NR3C1 gene:** NR3C1 (Glucocorticoid receptor, GR) (Glucocorticoid receptor; regulates HPA axis, stress response, inflammation, and metabolism); **SMARCA4 gene:** SMARCA4 (BRG1) (Chromatin-remodeling ATPase; epigenetic regulation of gene activity); **CpG site:** CpG dinucleotide (DNA regions; targets for methylation and epigenetic regulation); **RAGE receptor:** Receptor for Advanced Glycation End-products (Cell-surface receptor; triggers inflammatory and stress responses (implicated in diabetes, cancer, neurodegeneration)); **TLR:** Toll-like receptor (Pattern-recognition receptors; innate immune detection of PAMPs/DAMPs); **TNF:** Tumor necrosis factor (Inflammatory cytokine; regulates apoptosis and immune responses); **IFNγ:** Interferon gamma (Antiviral and immunomodulatory cytokine; supports Th1 responses); **MCC950:** MCC950 (Selective NLRP3 inflammasome inhibitor; designed to block excessive inflammation); **PAF:** Platelet-activating factor (Lipid mediator; modulates inflammation, thrombosis, and cell signaling); **DC:** Dendritic cell (Antigen-presenting cell; initiates adaptive immune responses); **Treg:** Regulatory T cell (Maintains immune regulation and tolerance); **ILC:** Innate lymphoid cell (Early immune responders; mucosal homeostasis and defense); **Teff:** Effector T cell (T-cell populations that execute immune responses (e.g., cytotoxic, helper)); **NIMA:** Nonhereditary maternal antigens (Maternal antigens not genetically inherited; relevant to immunological tolerance in pregnancy); **PRR:** Pattern recognition receptor (Receptors that detect PAMPs/DAMPs in innate immunity); **EVs:** Extracellular vesicles (Intercellular communication vehicles carrying proteins, RNAs, lipids); **DSC:** Decidual stromal cell (Uterine stromal cells involved in pregnancy, immune tolerance, and support); **HMGB1:** High mobility group box 1 (DNA-binding protein and DAMP; signals inflammation and cell stress); **HSP70:** Heat shock protein 70 (Chaperone protein; protein folding, stress protection, and immune modulation); **MC:** Microchimeric cell (Small cell group in development (context-dependent meaning)).
